# Local Perianal Anesthetic Infiltration Is Safe and Effective for Anorectal Surgery

**DOI:** 10.3389/fsurg.2021.730261

**Published:** 2021-09-09

**Authors:** Tomas Poskus, Matas Jakubauskas, Karolis Čekas, Lina Jakubauskiene, Kestutis Strupas, Narimantas Evaldas Samalavičius

**Affiliations:** ^1^Clinic of Gastroenterology, Nephrourology, and Surgery, Institute of Clinical Medicine, Faculty of Medicine, Vilnius University, Vilnius, Lithuania; ^2^Clinic of Obstetrics and Gynecology, Faculty of Medicine, Vilnius University, Vilnius, Lithuania; ^3^Department of Surgery, Klaipeda University Hospital, Klaipeda, Lithuania; ^4^Clinic of Internal, Family Medicine and Oncology, Institute of Clinical Medicine, Faculty of Medicine, Vilnius University, Vilnius, Lithuania; ^5^Health Research and Innovation Science Center, Faculty of Health Sciences, Klaipeda University, Klaipeda, Lithuania

**Keywords:** hemorrhoids, local anesthesia, anorectal surgery, sphincterotomy, hemorrhoidectomy

## Abstract

**Background:** General or regional anesthesia is predominantly used for anorectal surgery, however in the recent years more attention was drawn in the use of local anesthesia for anorectal surgery. In this study we present the technique and results of the use of local perianal anesthetic infiltration for minor anorectal operations.

**Methods:** In this cohort study patients undergoing surgery for hemorrhoids, anal fissures and low anal fistulas were included. Posterior perineal block was induced with a mixture containing 0.125% bupivacaine and 0.5% lidocaine. All patients were followed up at 30 days either by a post-operative visit or a telephone call and all post-operative complications over the post-operative 30-day period were registered.

**Results:** One thousand and twenty-six consecutive patients were included in our study. For all patients' intraoperative analgesia was achieved after performing perianal anesthetic infiltration and no additional support from the anesthesia team was necessary in any of case. Complications were observed in 14 (1.4%). Urinary retention occurred in 5 (0.5%) cases. Six cases of bleeding occurred after hemorrhoidectomy (0.6%) and 1 (0.1%) after lateral internal sphincterotomy. Perianal abscess developed for two patients (0.2%).

**Conclusions:** Local anesthesia using posterior perineal block technique is safe and effective for intraoperative analgesia in anorectal surgery, saving a substantial operation cost by avoiding the involvement of an anesthesia team and resulting in minimal incidence of urinary retention and other complications.

## Introduction

Hemorrhoidectomy, anal fistula surgery and lateral sphincterotomy make up a significant part of colorectal surgical practice in adult population. About 13.9 million (4–5%) people suffer from hemorrhoids and other anorectal disease in the USA and ~10% (1.4 million) of them require surgical intervention (1). Although these commonly performed anorectal operations are short in duration the dense sensory supply of the perineum leads to significant post-operative pain, making adequate anesthesia crucial (2). General or regional (spinal, caudal) anesthesia is predominantly used for anorectal surgery, however in the recent years several studies explored the use of local anesthesia for anorectal surgery (3–6). In this study we present the technique and results of the use of local perianal anesthetic infiltration for anorectal operations.

## Materials and Methods

### Study Population

Where applicable STROBE guidelines were employed to report this study (7). Patients, undergoing anorectal operations between July 2002 and July 2012, were enrolled in a prospectively collected and maintained database, noting their age, sex, indications for operation, performed operation and any complications within the 30 day after operation. Indications for operation were symptomatic third and fourth degree hemorrhoids (8), anal fissures after failed medical treatment, and low anal fistulas with no suspicion of upward extension and anal polyps. The indications for operation and operative tactics were in line with the current colorectal surgery guidelines (9–11). Exclusion criteria included complicated anal pathologies (incontinence, stenosis, or abscess), other comorbidities (inflammatory bowel diseases, acquired immune deficiency syndrome or tuberculosis), documented allergy to local anesthesia or patient unwillingness to undergo local anesthesia. The study was approved by the bioethics committee and all patients were informed about the technique of the procedure and detailed written consent was obtained beforehand.

### Pre-operative Preparation and Local Anesthesia Technique

**Figure 1:**
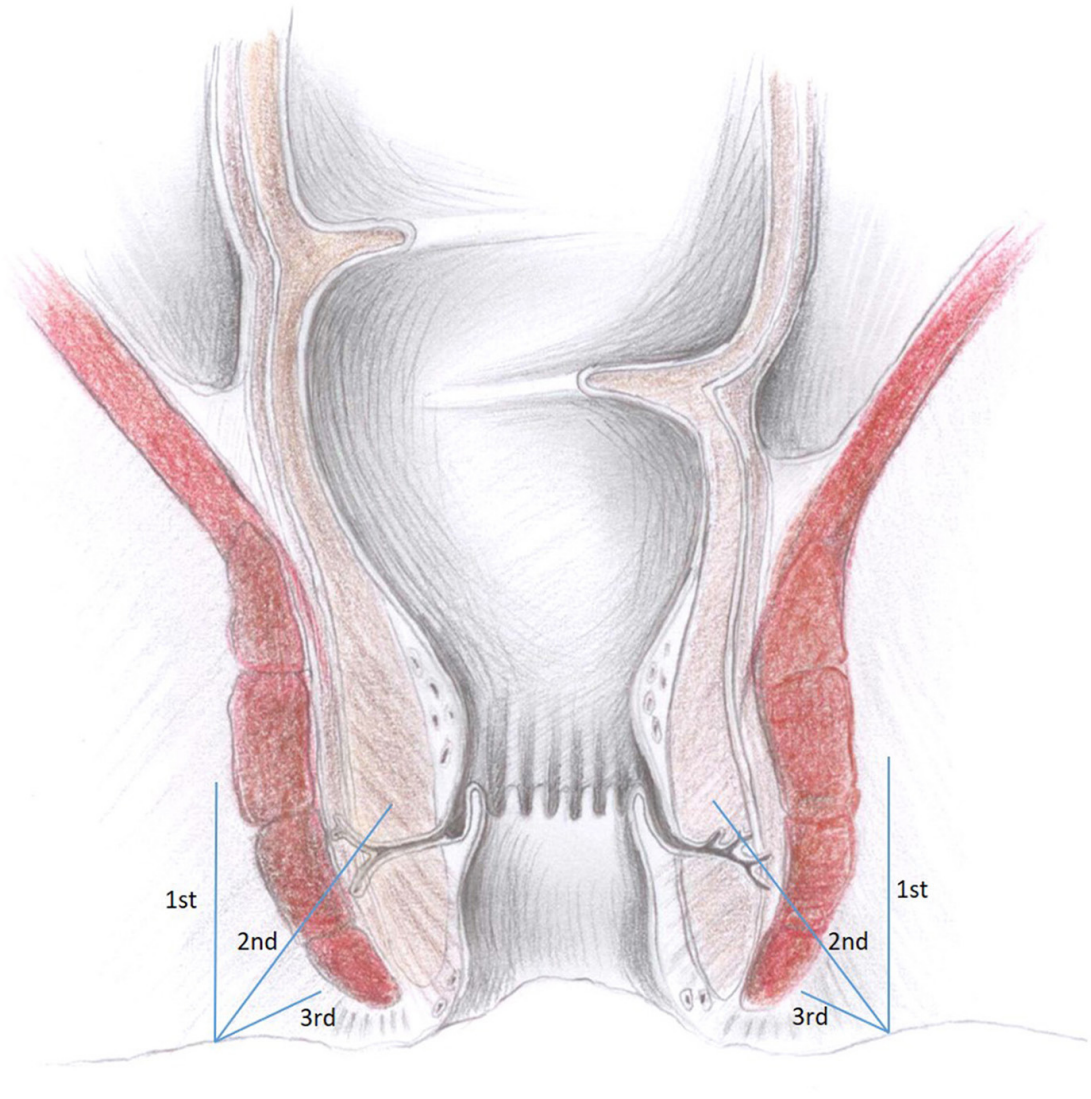
Directions of all anesthetic injections visualized in the frontal plane.

**Figure 2:**
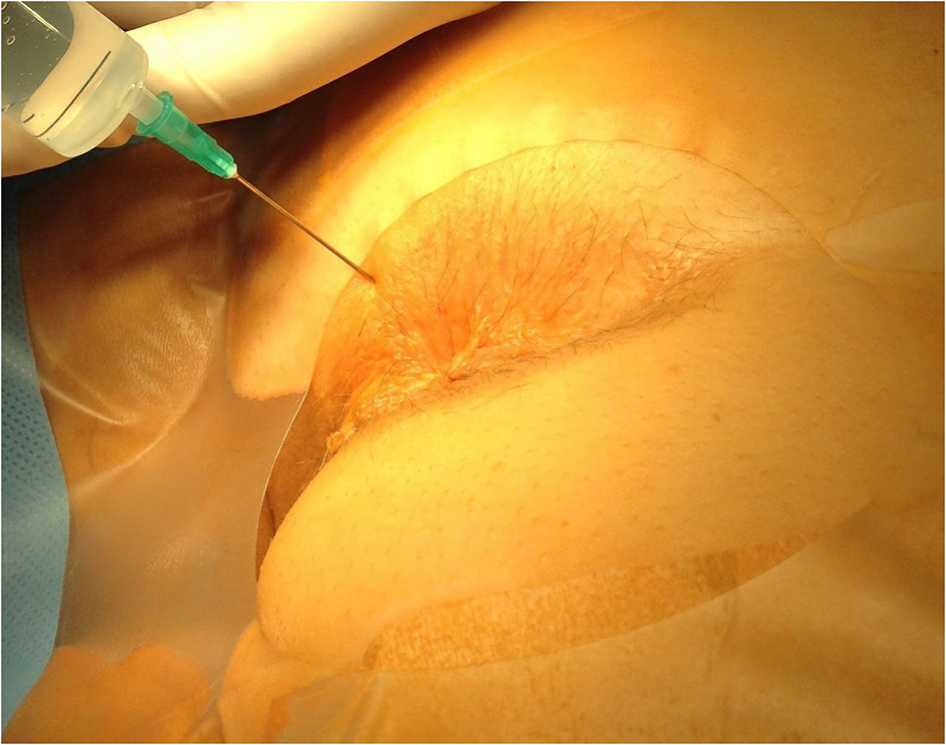
Direction of the first injection.

**Figure 3:**
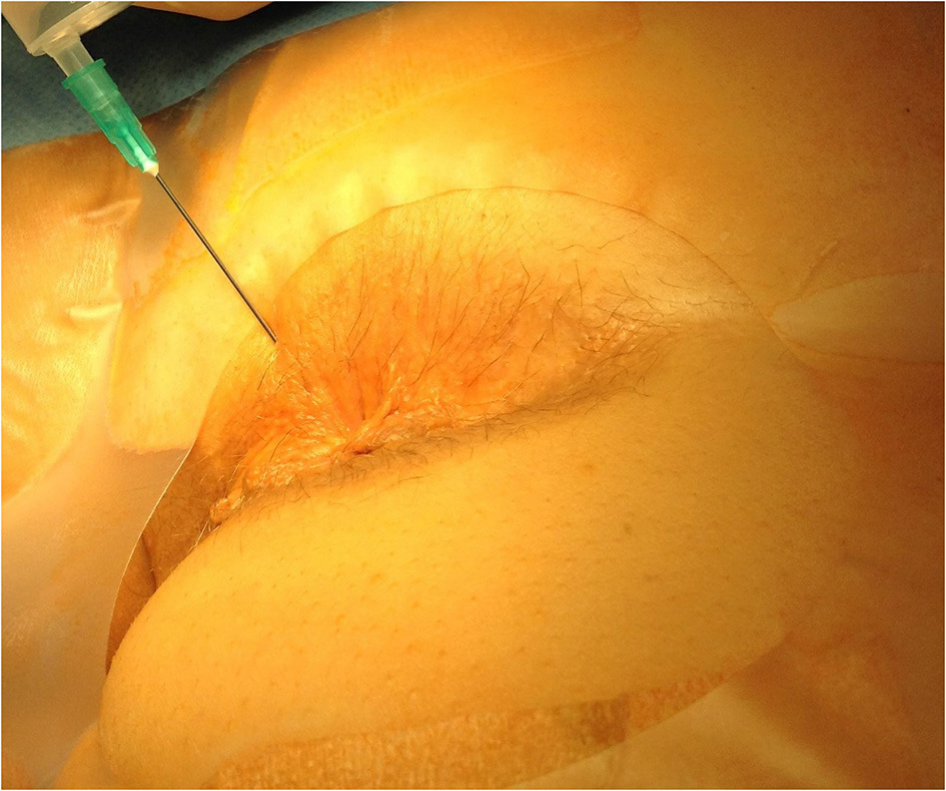
Direction of the second injection.

**Figure 4:**
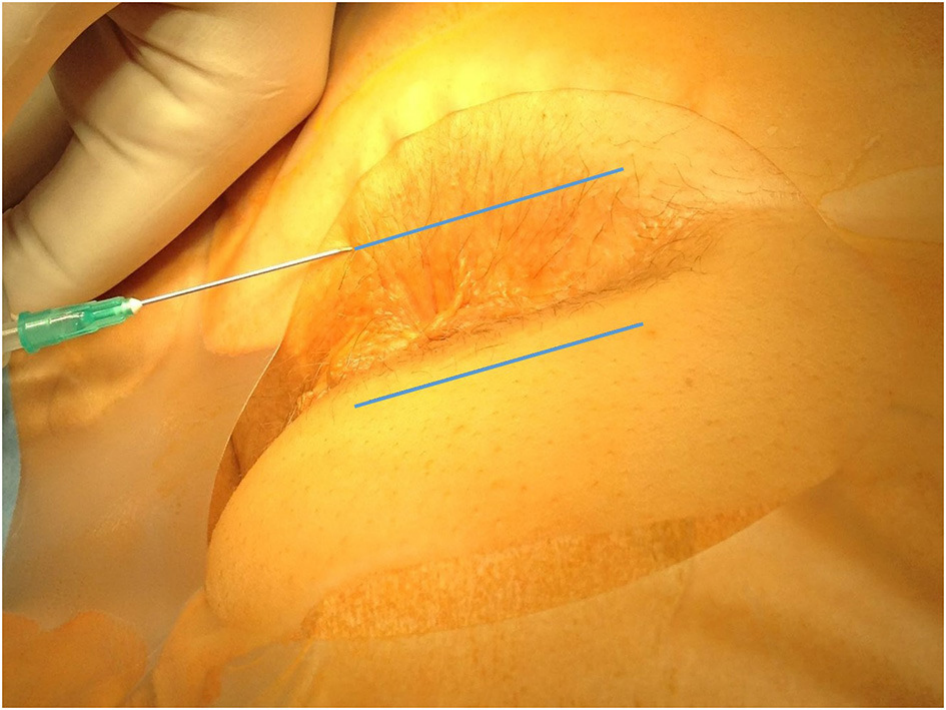
Direction of the third injection (line marks the bearing of the subcutaneous injection).

The anesthesia technique was learned from Lohsiriwat D (personal communication). All patients received lactulose pre-operatively and no bowel preparation was used. No intravenous or oral sedation was used and no anesthesia team was present in the operating room. Electrocardiography, pulse oxymetry and blood pressure monitoring was used in every case. In all cases patients were placed in the prone jackknife position. Posterior perineal block was induced with 42 ml of mixture containing 0.125% bupivacaine and 0.5% lidocaine. Three consecutive injections through one skin puncture site on each side of the anus were performed, with skin puncture points being anteriorly 2–2.5 cm from the dentate line on the skin and 1.5–2 cm from the midline. Each of the three 7 ml injections was pointed at different directions (Figure 1). The first was parallel and external to the anal sphincter complex (Figure 2). The second was performed at a 45 degrees angle to the skin, aiming at the top midline of the anal canal (Figure 3). The third was performed subcutaneously, parallel to the skin surface (Figure 4). Skin infiltration was avoided. Injections were performed after aspiration test confirmed that the needle was not in the lumen of the vessel. Same sequence of injections was repeated contralaterally.

### Surgical Techniques

Closed hemorrhoidectomy was used for hemorrhoids. Only symptomatic cushions were removed. Internal hemorrhoids in remaining untreated locations were coagulated by bipolar coagulation. Tailored closed lateral internal sphincterotomy was performed for medically untreatable anal fissure. Internal anal sphincter was incised to the level of the dentate line. Only patients, who had low and simple anal fistulas when there was no suspicion of upward extension were treated under posterior perineal block anesthesia. In such cases, fistulotomy with laying open of the fistula track was performed. Anal polyps were simply excised using electrocautery. No wound or anal packing was used.

### Post-operative Management and Follow-Up

Patients were discharged on the day of operation if they were able to urinate, if the pain was under control with oral pain medications and if the social circumstances were favorable (support at home, no need to travel far after the operation). All patients were followed up at 30 days either by a post-operative visit or a telephone call and all post-operative complications over the post-operative 30-day period were registered.

Study results are presented as absolute numbers and percentages for categorical variables and as medians with ranges for continuous variables.

## Results

One thousand and twenty-six consecutive patients, with the median age of 48 (range 17–89) years, underwent operations for anorectal diseases from July 2002 to July 2016. Of them, 494 (48.1%) were male and 532 (51.9%) female. For all patients' intraoperative analgesia was achieved after performing perianal anesthetic infiltration and no additional support from the anesthesia team was necessary in any of case. Hemorrhoidectomy was performed in 835 (81.4%) cases (Table 1). Of them, 100 (12%) patients had simultaneous operations: lateral internal sphincterotomy for concomitant anal fissure in 72 (72.0%), anal polypectomy in 23 (23.0%) and fistulotomy for low fistula in 5 (5.0%) patients.

**Table 1:** Performed surgical procedures.

**Surgical procedure**	**Number of cases [*n* (%)]**
Hemorrhoidectomy	835 (81.4)
**Number of cushions removed**	
One	318 (31.0)
Two	227 (22.1)
Three	283 (27.6)
Four	7 (0.7)
**Grade**	
III	452 (54.1)
IV	383 (45.9)
Closed lateral internal anal sphincterotomy	162 (15.8)
Fistulotomy	15 (1,5)
Anal polypectomy	14 (1,4)

Closed lateral internal sphincterotomy was performed for chronic anal fissure in 162 (15.8%) patients. Of them, in 25 (15.4%) cases simultaneous procedures were performed: 7 (28.0%) anal polypectomies, 7 (28.0%) fistulotomies for low anal fissures and in 11 (44.0%) cases internal hemorrhoids were coagulated with bipolar coagulation or ligated.

Complications were observed in 14 (1.4%) patients (Table 2). Urinary retention occurred in 5 (0.5%) cases, requiring placement of urinary catheter. Six cases of bleeding occurred after hemorrhoidectomy (0.6%) and 1 (0.1%) after lateral internal sphincterotomy. In two cases bleeding occurred within first 2 h after the operation and it was stopped by oversewing of the bleeding spot without any additional anesthesia. Other five patients had to be repeatedly anesthetized with posterior perineal block and underwent a thorough surgical wound hemostasis.

**Table 2:** Post-operative complications.

**Complications**	**Number of cases [*n* (%)]**
Hemorrhage	7 (0.7)
Urinary retention	5 (0.5)
Perianal abscess	2 (0.2)

Perianal abscess developed for two patients (0.2%): in one case after hemorrhoidectomy and in the other after a sphincterotomy. Abscesses occurred within 2 weeks after the operation and manifested with fever and increasing perianal pain. In both cases they required surgical drainage and resolved completely. Seven hundred and fifty-three (73.4%) patients underwent a day-care procedure. The median hospital stay was 1.8 days (1–18 days).

## Discussion

Our study suggests that local anesthesia using the posterior perineal block technique ensures safe and effective intra-operative and post-operative analgesia for most commonly performed anorectal operations.

Other studies also confirmed that patients have virtually no complications after posterior perianal block and that this anesthesia technique is easy to perform, can be safely applied by any surgeon, potentially reduce operation costs, is associated with a shorter hospital stay and ensure a faster patients return to full social activities (1, 3, 4, 12–15).

However, this anesthesia technique has some disadvantages. One of them is the inadequate relaxation of the puborectalis muscle (12). Therefore, patients with high perianal fistulas or adenomas higher in the rectum, cannot be operated upon using this anesthesia technique. Also ambulatory anorectal surgery has a limited time for direct post-operative observation of the patient.

The main limitations of the study are the lack of objective pain measuring and no comparison with other anesthesia techniques. Furthermore, this is a descriptive type of study that lacks a thorough statistical analysis, which could help to draw more robust conclusions. However, our study included quite a large consecutive cohort of unselected patients undergoing different anorectal operations.

During this type of operations patient positioning is important. In most practices lithotomy or jackknife positions are preferred. Lithotomy position is usually preferred by the anesthesiologist, who controls the airways, but it is awkward for the surgeon, as patients' buttocks may obscure vision and manipulation (4). Alternatively, perianal anesthetics infiltration permits the use of a safe jack-knife position, which is convenient in having good exposure of the operative field and direction of injection (3, 4, 16).

According to studies, the most commonly used local anesthetics for such implications are lidocaine, bupivacaine, mepivacaine (3–5, 16). In our study local anesthesia was induced with a mixture of 0.125% bupivacaine and 0.5% lidocaine. Lidocaine is a short-acting local anesthetic which provides an excellent initial pain relief, whereas bupivacaine is a long-acting anesthetic providing several hours of anesthesia post-operatively (17). Some surgeons add adrenaline to the anesthetic, which promotes vasoconstriction and reduces bleeding in the operative field (3). Unfortunately, we were unable to find any studies directly comparing different local anesthetics for anorectal surgery.

We observed quite a low (1.4%) post-operative complication rate in our study. One of the most common complications after anorectal operations is urinary retention (18). It is mostly related to spinal anesthesia, fluid overload and post-operative pain (19, 20). Spinal or caudal anesthesia and pudendal (ischiorectal) nerve blocks may cause urinary retention in up to 36% of patients (1, 12, 21). The reported rate of urinary retention after general anesthesia is around 3% (1, 6). The use of perianal infiltration of local anesthetics allows anorectal surgery to be performed with a very low incidence of urinary retention (3, 21). We report a 0.5% rate of urinary retention in our study, which is very similar to the rates, ranging from 0 to 0.5%, reported by other studies (3–5, 12, 16, 21).

The rate of post-operative bleeding was reported to be up to 3% after general, 12% after regional and from 0.5 to 8% after local anesthesia (1, 3, 16, 21). Few studies have also reported zero bleeding rates after local anesthesia (4, 5, 12). In our study the rate of post-operative bleeding was 0.7%. We think that the surgical technique with meticulous hemostasis and selection of the patients with normal coagulation parameters are more important in preventing post-operative bleeding, rather than the method of anesthesia.

Since hemorrhoidectomy wounds rarely heal primarily, the true rate of wound infection is unknown, however, the instances of perianal abscess after surgery are reported. Wound infection rate under spinal anesthesia was reported to be up to 4% (21). Local anesthesia studies report almost no cases of wound infection and only two patients (0.2%) in our study developed perianal abscesses (3, 4, 12, 21).

Overall current literature indicates that local anesthesia is safe and even in some cases superior to spinal anesthesia for anorectal surgical procedures.

The reported high patient satisfaction with local anesthesia may be related to the short hospital stay and adequate control of intra-and post-operative pain. The success of the local anesthetics technique is also highly dependent on the skills of the surgeon in providing effective infiltration (3). Specific post-operative recommendations, which include a high residual diet, potent oral analgesics, mild laxative drugs and a warm sitz bath, may help to further increase patient satisfaction after anorectal surgery (14).

## Conclusion

Local anesthesia using posterior perineal block technique is safe and effective for intraoperative analgesia in anorectal surgery, saving a substantial operation cost by avoiding the involvement of an anesthesia team and resulting in minimal incidence of urinary retention and other complications.

## Data Availability Statement

The raw data supporting the conclusions of this article will be made available by the authors, without undue reservation.

## Ethics Statement

The studies involving human participants were reviewed and approved by Vilnius Regional Bioethics Committee. The patients/participants provided their written informed consent to participate in this study.

## Author Contributions

TP, KS, and NS: conceptualization, methodology, resources, writing-review and editing, and supervision. MJ, KČ, and LJ: software and data curation. TP, MJ, KČ, and LJ: validation. TP, MJ, and NS: formal analysis. TP, MJ, KČ, LJ, KS, and NS: investigation. TP, MJ, and KČ: writing-original draft preparation. TP and MJ: visualization. All authors contributed to the article and approved the submitted version.

## Conflict of Interest

The authors declare that the research was conducted in the absence of any commercial or financial relationships that could be construed as a potential conflict of interest.

## Publisher's Note

All claims expressed in this article are solely those of the authors and do not necessarily represent those of their affiliated organizations, or those of the publisher, the editors and the reviewers. Any product that may be evaluated in this article, or claim that may be made by its manufacturer, is not guaranteed or endorsed by the publisher.
